# Assessment of Corneal Stromal Remodeling and Regeneration after Photorefractive Keratectomy

**DOI:** 10.1038/s41598-018-30372-2

**Published:** 2018-08-22

**Authors:** Pouriska B. Kivanany, Kyle C. Grose, Madhavi Tippani, Shan Su, W. Matthew Petroll

**Affiliations:** 1 Department of Ophthalmology, UT Southwestern Medical Center, Dallas, TX USA; 2 Biomedical Engineering Graduate Program, UT Southwestern Medical Center, Dallas, TX USA

## Abstract

This study utilizes high resolution multi-dimensional imaging to identify temporal and spatial changes in cell/extracellular matrix (ECM) patterning mediating cell migration, fibrosis, remodeling and regeneration during wound healing. Photorefractive keratectomy (PRK) was performed on rabbits. In some cases, 5([4,6-dichlorotriazin-2yl]-amino)fluorescein (DTAF) was applied immediately after surgery to differentiate native vs. cell-secreted collagen. Corneas were assessed 3–180 days postoperatively using *in vivo* confocal microscopy, and cell/ECM patterning was evaluated *in situ* using multiphoton and second harmonic generation (SHG) imaging. 7 days post-PRK, migrating fibroblasts below the ablation site were co-aligned with the stromal lamellae. At day 21, randomly patterned myofibroblasts developed on top of the ablation site; whereas cells underneath were elongated, co-aligned with collagen, and lacked stress fibers. Over time, fibrotic tissue was remodeled into more transparent stromal lamellae. By day 180, stromal thickness was almost completely restored. Stromal regrowth occurred primarily below the ablation interface, and was characterized by co-localization of gaps in DTAF labeling with elongated cells and SHG collagen signaling. Punctate F-actin labeling was detected along cells co-aligned with DTAF and non-DTAF labeled collagen, suggesting cell-ECM interactions. Overall, collagen lamellae appear to provide a template for fibroblast patterning during wound healing that mediates stromal repopulation, regeneration and remodeling.

## Introduction

Corneal opacification is a leading cause of blindness worldwide^1^. Opacification can occur from various sources, such as injury, infection, chemical burns, or surgery. Following injury, surgery or other insults, corneal keratocytes can become activated by growth factors and other cytokines present in the wound environment, and transform into a fibroblastic phenotype^2,3^. Corneal fibroblasts proliferate, develop intracellular stress fibers, and migrate into the wound. In certain wound types, the presence of transforming growth factor beta (TGFβ) in the wound can induce transformation of corneal fibroblasts to myofibroblasts, which generate stronger forces on the matrix and synthesize a disorganized fibrotic ECM^4,5^. Together, these processes can impact visual acuity by altering corneal shape and reducing transparency due to increased light scattering by both cells and the newly synthesized ECM^6–11^. Even routine corneal procedures, such as photorefractive keratectomy (PRK) and laser assisted *in situ* keratomileusis (LASIK) can lead to fibrosis in about 2–4% of eyes, and the chance of developing haze is proportional to the correction level needed^12–18^. Haze formation can greatly affect the quality of life for patients; thus, there is a need for therapies that can inhibit the initial development of fibrotic tissue after corneal injury or surgery, or stimulate remodeling of pre-existing fibrotic tissue or scars into transparent tissue.

Previous studies have shown that following keratectomy surgery in the rabbit, there is remodeling of fibrotic tissue and regeneration of stromal tissue over time^19–21^. Studies by Jester and coworkers showed that following PRK in the rabbit, there was an initial fibrotic response at 21 days which resulted in significant corneal haze. Over time, however, this fibrotic tissue was remodeled, and by 17 weeks, corneal transparency was restored^20,21^. In addition, regrowth of the corneal stroma under the wound bed resulted in a gradual return towards pre-operative thickness. Cell and extracellular matrix (ECM) mechanical interactions and patterning play an important role in the development and maintenance of corneal transparency, the response of the cornea to injury or refractive surgery, and the structural organization of tissue engineering constructs^21,22^. Feedback from ECM (topography, stiffness) has been increasingly recognized as a key regulator of the biochemical signaling pathways that drive cell differentiation into diverse phenotypes, and the alignment of cells and the forces they generate has been shown to impact collagen deposition, organization and alignment *in vitro*^23–27^. However, a detailed temporal and spatial assessment of the changes in cell/ECM interactions and patterning that mediate long term corneal stromal remodeling and regeneration *in vivo* has not been reported.

In this study, we address this gap by using a combination of high resolution 3-D imaging techniques including *in vivo* confocal microscopy, *in situ* multiphoton fluorescence imaging, and second harmonic generation (SHG) imaging to assess changes in cell and matrix patterning during wound healing following PRK in the rabbit. By using en face imaging *in situ* combined with DTAF labeling to distinguish native versus secreted collagen, we simultaneously assess cell and lamellar patterning during all four phases of wound healing (migration, fibrosis, remodeling, regeneration) for the first time. We also track and quantify regeneration (stromal growth), calculate stromal cell-ECM co-alignment, and use specific protein markers to characterize stages of wound healing over time.

## Results

### *In Vivo* Assessment

**Figure 1:**
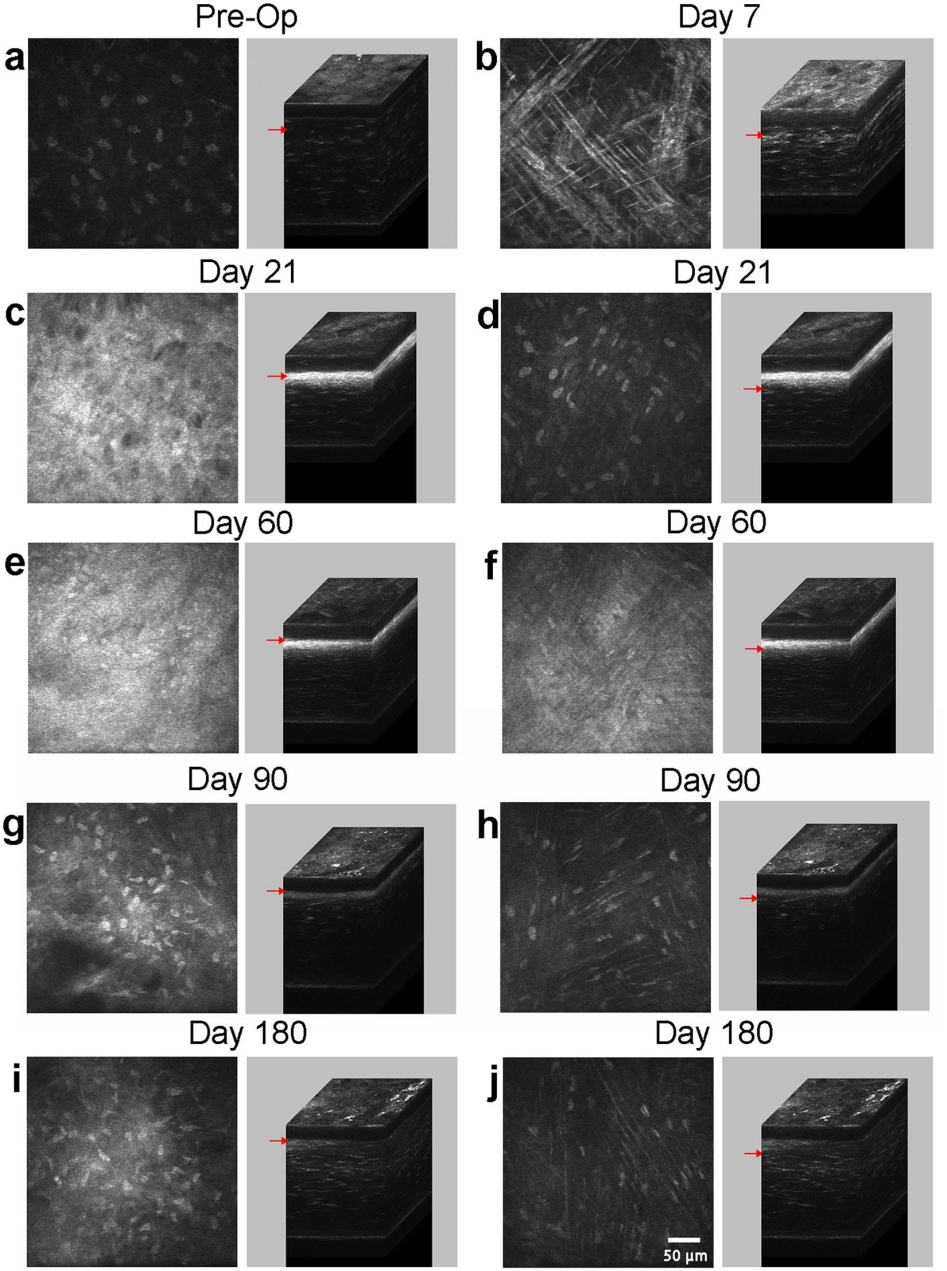
*In vivo* 2-D confocal images (left) with corresponding 3-D reconstruction of scans (right). Representative images from the (**a**) pre-operative (Pre-Op) cornea, (**b**) 7 days after PRK, (**c**,**d**) 21 days after PRK, (**e**,**f**) 60 days after PRK, (**g**,**h**) 90 days after PRK, and (**i**,**j**) 180 days after PRK. Images (**a**,**b**) are from the anterior stroma, (**c**,**e**,**g**,**i**) are from the *fibrosis/remodeling* region on top of the photoablated surface, and images (**d**,**f**,**h**,**j**) are located in the *regenerative* region just below the photoablated surface. Arrows indicate the location of each 2-D image within the 3-D stack.

Representative 2-D and 3-D confocal microscopy through focusing (CMTF) images are shown in Fig. 1. In the normal cornea, backscatter of light in the stroma came primarily from the keratocyte nuclei (Fig. 1a). After PRK, a region of cell death is created under the photoablated surface, which was observed at day 3 (not shown)^7,28^. At 7 days, this region was repopulated by elongated and reflective cells that were often co-aligned (hereafter referred to as the *migrating* region; Fig. 1b). By day 21, stromal haze was at a maximum, and two distinct patterns of cells were observed (Fig. 1c,d). Cells anterior to the photoablated surface (hereafter referred to as the *fibrosis/remodeling* region) were dense, interconnected in a random pattern, and highly reflective (Fig. 1c). Directly posterior to the photoablated surface (hereafter referred to as the *regenerative* region), cells within the stroma were thin, elongated and organized into parallel groups, and did not appear as reflective (Fig. 1d). By day 60, cells in the *fibrosis/remodeling* region were reduced in reflectivity (Fig. 1e), whereas cells in the *regenerative* region remained elongated and co-aligned (Fig. 1f). By day 90 and 180, cellular backscatter was limited to the keratocyte nuclei in the *remodeling* region (Fig. 1g,i), indicating a more normal quiescent phenotype. However, diffuse haze was observed between cells (compare Fig. 1g,h with Fig. 1a). Cells in the *regenerative* region remained highly aligned with reduced backscatter as compared to day 60 (Fig. 1h,j). Overall stromal reflectivity also appeared to decrease at day 90 and 180 (Fig. 1g–j; 3D reconstruction of cornea), as compared to day 21 (Fig. 1c,d; 3D reconstruction of cornea).

**Figure 2:**
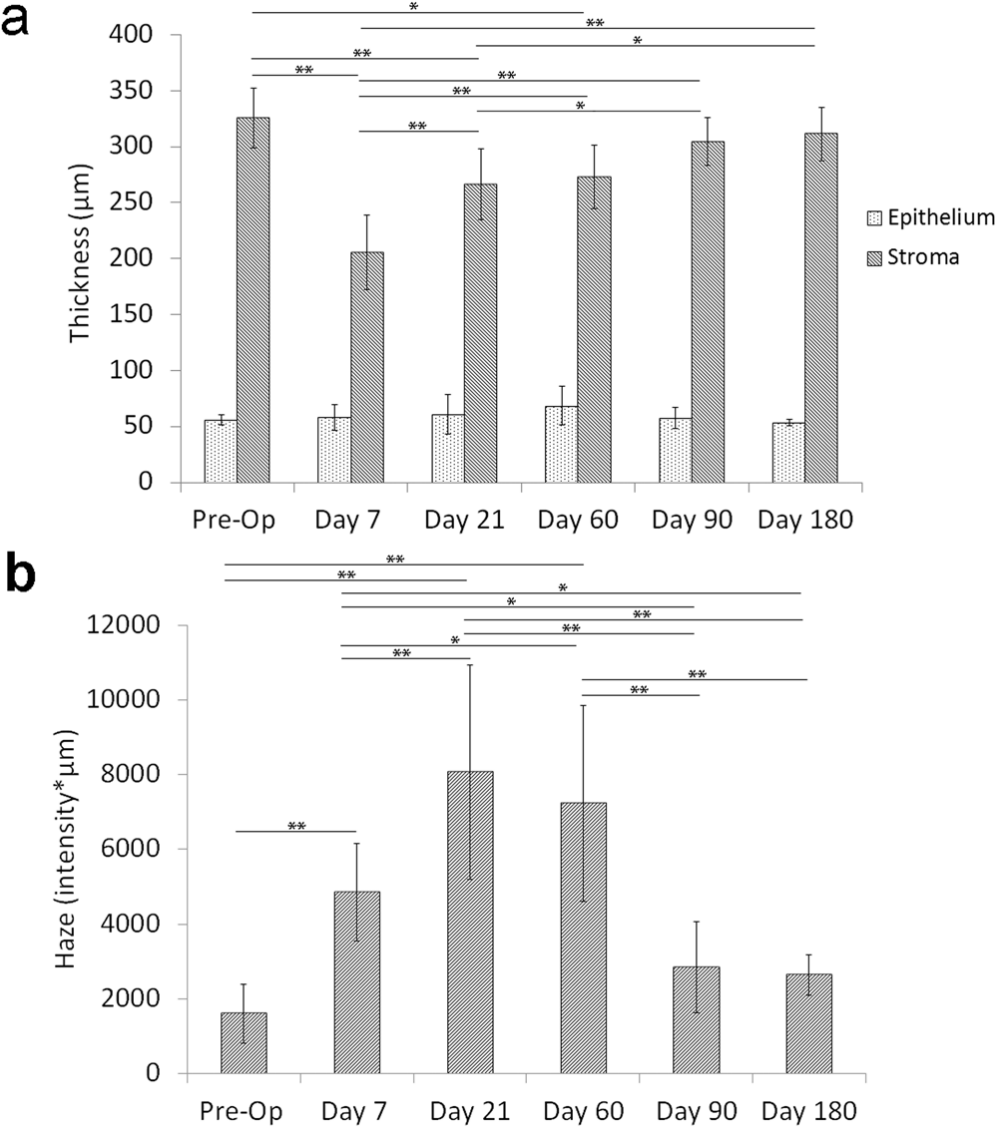
Measurements from *in vivo* confocal scans. (**a**) Epithelial and stromal thickness at each time point. (**b**) Stromal haze calculated at each time point. (ANOVA between groups, *P < 0.05, **P < 0.001).

Quantitative CMTF analysis is shown in Fig. 2. By day 7, the epithelium had returned to normal thickness (Fig. 2a). Stromal thickness initially decreased by over 110 µm, as a result of the PRK procedure (day 7); however, between day 7 and day 180, stromal thickness gradually increased back to 94.5% ± 7.5 of the pre-operative value. Haze values were increased at 7 days following PRK, with maximum haze values calculated at day 21 (Fig. 2b). Haze values started to decline by day 60, and decreased further to a near normal value by day 90. Although not statistically significant, haze was still slightly elevated at days 90 and 180, due to the diffuse ECM backscatter in the remodeled region (Fig. 1g,i).

Prior to CMTF scanning, rabbit corneas were qualitatively assessed using slit lamp microscopy (Supplementary Fig. 1). In the unoperated eye, corneas appeared transparent without haze (Supplementary Fig. 1). At day 21, prominent haze was observed in the central cornea corresponding the PRK location in all eyes assessed (Supplementary Fig. 1). By day 90 (Supplementary Fig. 1), there was little to no corneal haze seen from slit lamp examinations in all eyes assessed.

### Immunocytochemistry

**Figure 3:**
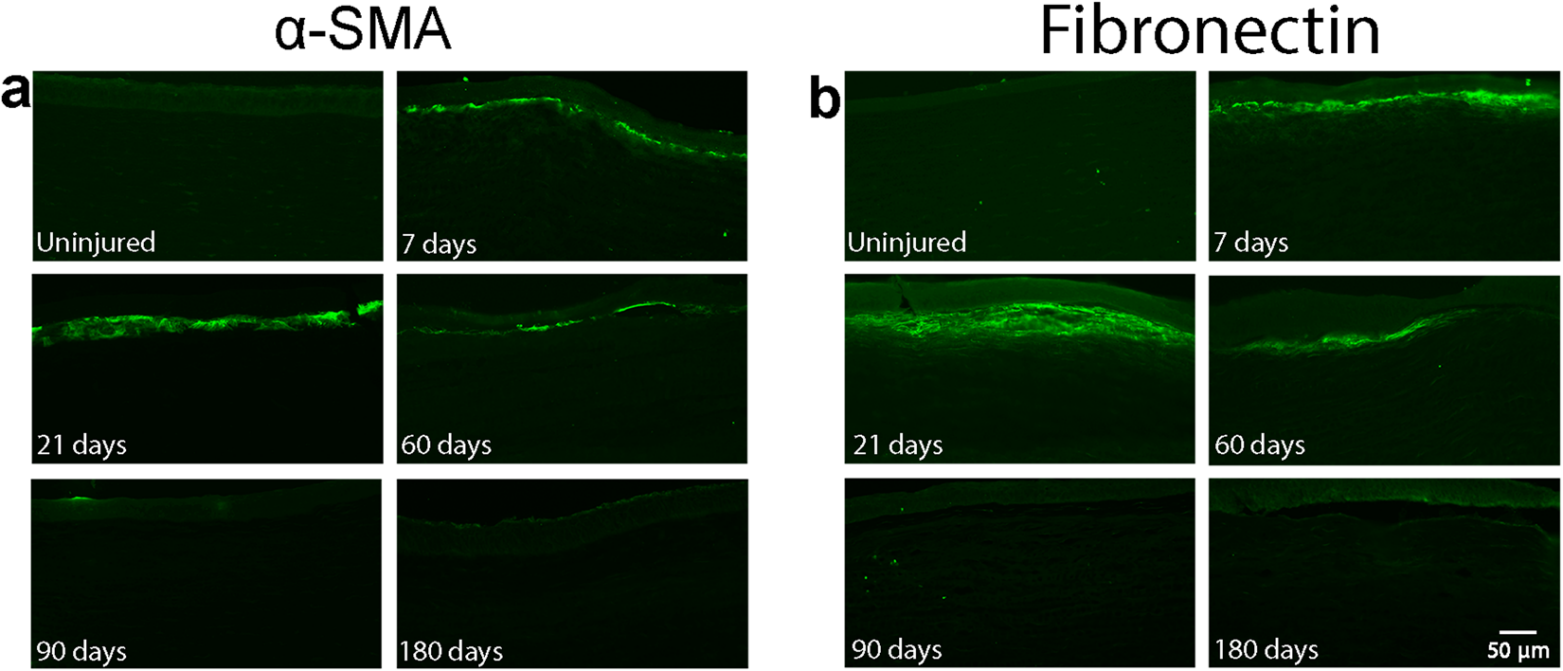
Immunohistochemistry for detection of α-SMA and fibronectin near the center of the photoablated region. (**a**) Contains images from frozen sections labeled for α-SMA, and (**b**) contains images from frozen sections labeled for fibronectin at each time point. Images are from the anterior segment of the cornea.

At 7 days following PRK, there was only weak detection of α-SMA (Fig. 3a) and fibronectin (Fig. 3b) in the wound area in corneal cross-sections. At 21 days, there was a drastic increase in α-SMA and fibronectin. By 60 days, α-SMA and fibronectin was reduced in the wounded region of the cornea, and by day 90 and 180, no α-SMA or fibronectin was detected. Note that when detected, fibronectin and α-SMA was always on top of the DTAF-labeled native stromal tissue (not shown).

### *In Situ* Multiphoton and Second Harmonic Generation (SHG) Imaging

**Figure 4:**
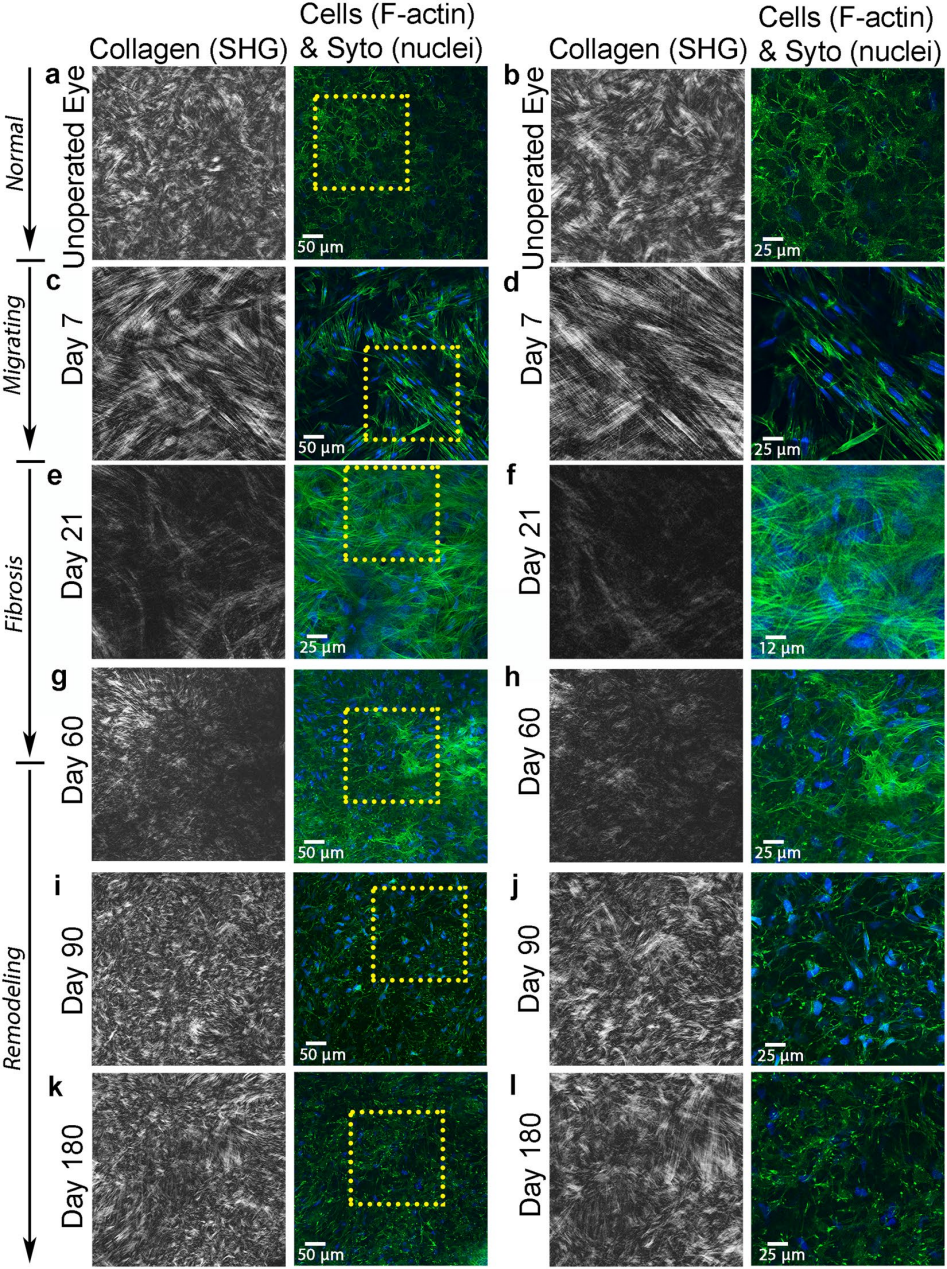
SHG forward scatter (collagen) and multiphoton fluorescent (F-actin-green, and nuclei-blue) en face images. Images are from: (**a**,**b**) unoperated cornea, and (**c**,**d**) 7 days, (**e**,**f**) 21 days, (**g**,**h**) 60 days, (**i**,**j**) 90 days, and (**k**,**l**) 180 days after PRK. Images a, b were taken 20 µm posterior to the basal lamina, and c, d were collected 24 µm posterior to the photoablation site in the *migrating* region. Images e-l were collected from the *fibrosis*/*remodeling* region on top of the photoablated surface. Images (**b**,**d**,**f**,**h**,**j**,**l**) are 2x zoomed-in (yellow box) images from (**a**,**c**,**e**,**g**,**i**,**k**), respectively.

**Figure 5:**
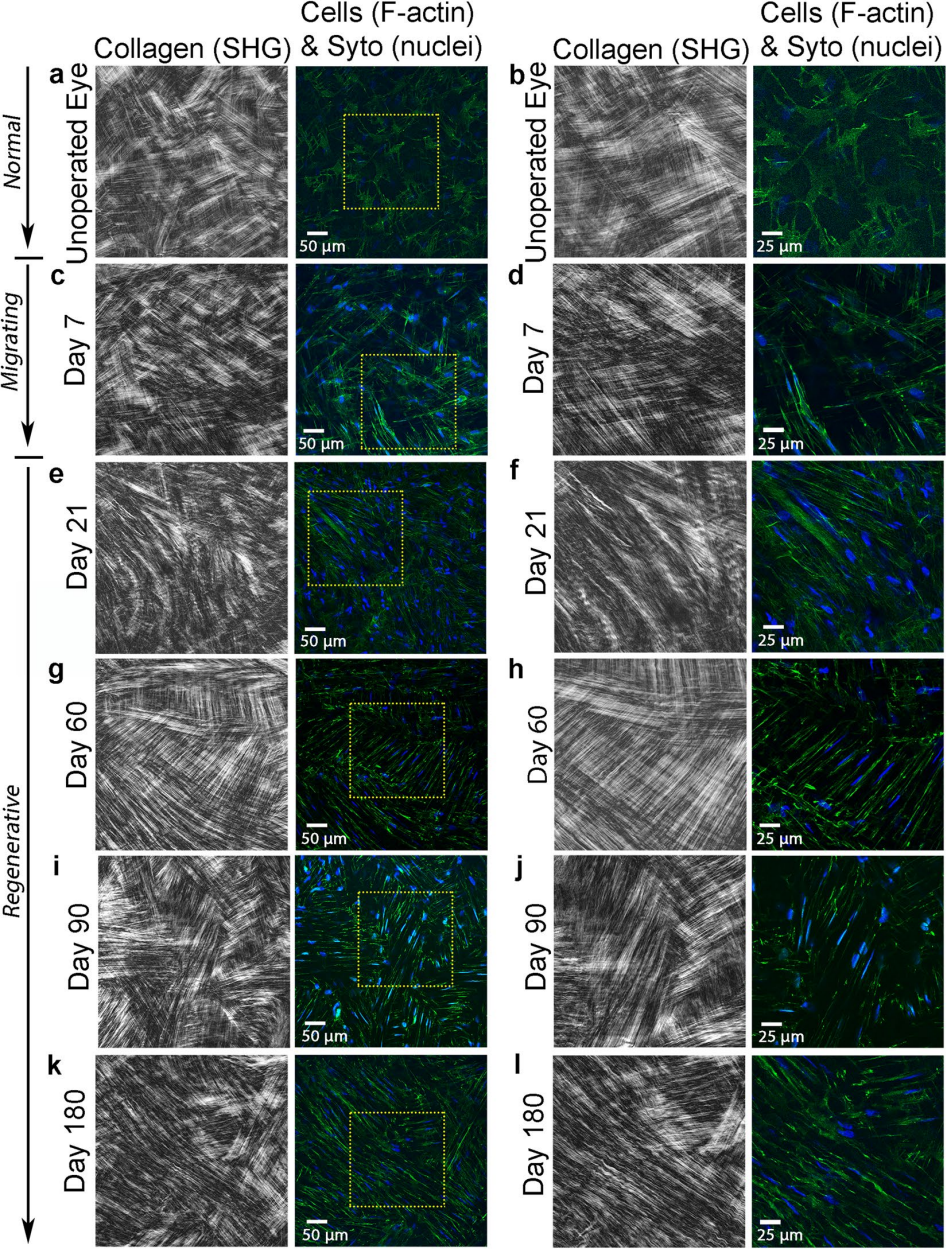
SHG forward scatter (collagen) and multiphoton fluorescent (F-actin-green, and nuclei-red) en face images. Images are from: (**a**,**b**) unoperated cornea, and (**c**,**d**) 7 days, (**e**,**f**) 21 days, (**g**,**h**) 60 days, (**i**,**j**) 90 days, and (**k**,**l**) 180 days after PRK. Images (**a**,**b**) were taken 128 µm posterior to the basal lamina, and c, d were collected 40 µm posterior to the photoablation site in the *migrating* region. Images e-l were collected from the *regenerative* region below the photoablated surface. Images (**b**,**d**,**f**,**h**,**j**,**l**) are 2x zoomed-in (yellow box) images from (**a**,**c**,**e**,**g**,**i**,**k**), respectively.

In the unoperated eye, collagen in the anterior stroma within 20 microns of the epithelium appeared highly interwoven in *en face* SHG images, and cells were quiescent with stellate morphologies, characteristic of normal keratocytes (Fig. 4a,b). Wider lamellae with less interweaving were observed deeper within the stroma (Fig. 5a,b; taken about 128 µm below the basal lamina to allow comparison with stroma under photoablated region)^21,29^. By 7 days, keratocytes from the wound margin had transformed into fibroblasts and migrated to the central cornea; these fibroblasts were characterized by elongated morphologies and more prominent F-actin labeling (Figs 4c,d and 5c,d). Migrating fibroblasts appeared to be co-aligned with the collagen lamellae and interconnected within parallel groups.

At day 21, myofibroblasts in the *fibrosis/remodeling* region on top of the photoablated surface contained prominent stress fibers, and were broader and more randomly arranged (Fig. 4e,f). There was no prominent fibrillar collagen (Fig. 4e,f) observed by SHG imaging; however, immunolabeling demonstrated that type I collagen was present in this region (Supplementary Fig. 2). By day 60, collagen in the *fibrosis/remodeling* region appeared to have an interwoven pattern, and cells appeared more quiescent in some areas (Fig. 4g,h). At day 90 and 180, cells throughout the *remodeling* region were quiescent, and collagen was highly interwoven (Fig. 4i–l, respectively), similar to the normal subepithelial stroma (Fig. 4a,b).

In contrast to the *fibrotic/remodeling* region, collagen within the *regenerative* region underneath the photoablated surface remained highly aligned in a lamellar pattern throughout the wound healing process (Fig. 5e–l), similar to the normal cornea (Fig. 5a,b). Cells in the regenerative region were elongated and co-aligned with the collagen, and stress fibers were not observed.

**Figure 6:**
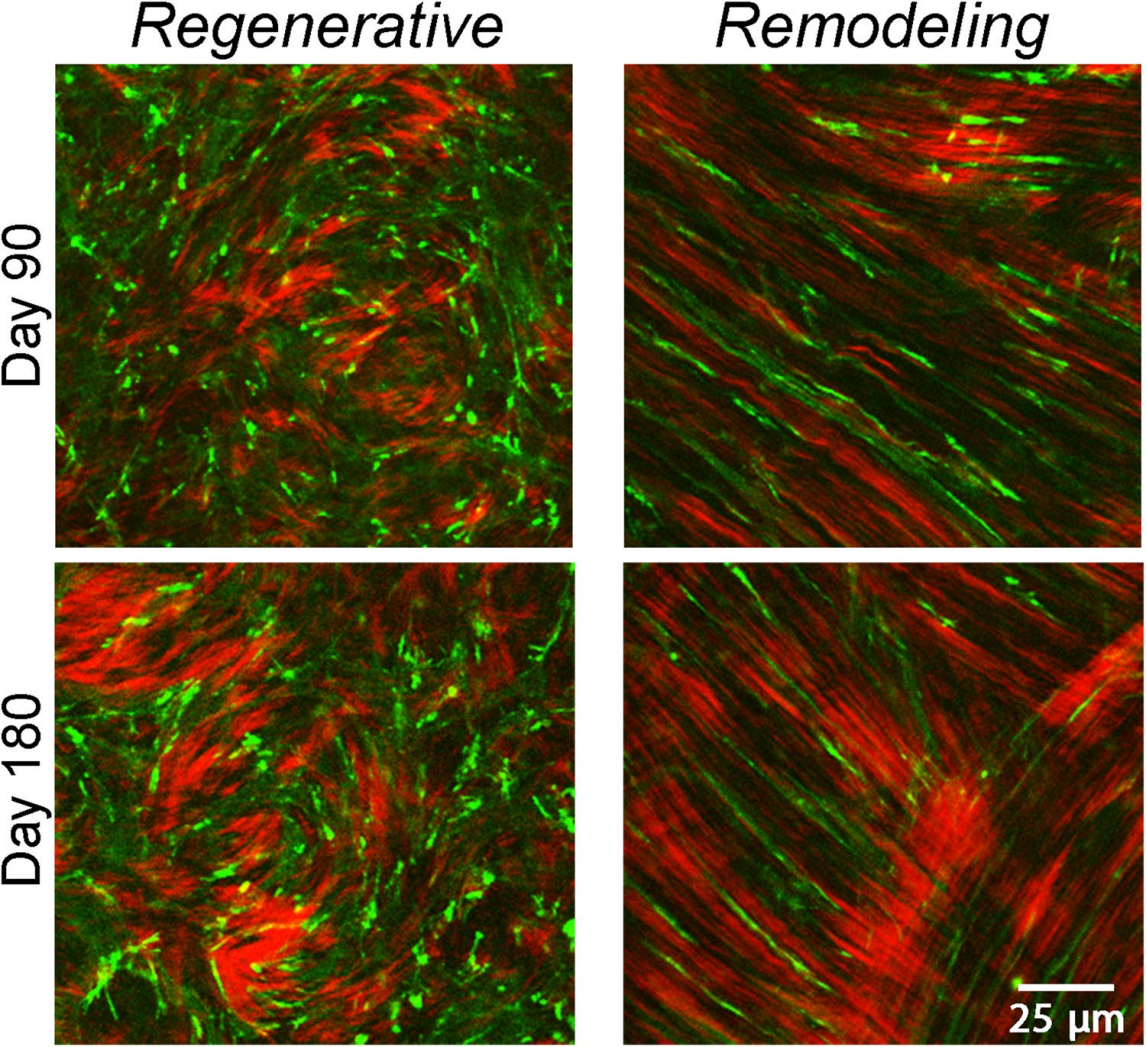
High magnification SHG and multiphoton image overlays showing association between collagen (shown in red) and F-actin (shown in green) during stromal remodeling and regeneration. Note that the pattern of punctate F-actin labeling is highly correlated with the collagen, suggesting cell-matrix interactions.

From day 7–180, bright, punctate F-actin labeling was often observed within cells (Figs 4d,h,j,l and 5d,f,h,j,l). High magnification overlays of F-actin and forward scattered SHG images demonstrate that the localization and alignment of punctate F-actin labeling corresponded to the pattern of collagen organization in both the *remodeling* and *regenerative* regions (Fig. 6). Punctate spots were often co-localized with collagen fibers suggesting possible sites of cell-ECM contact. This labeling pattern was most prominent on from days 60 to 180 after surgery.

**Figure 7:**
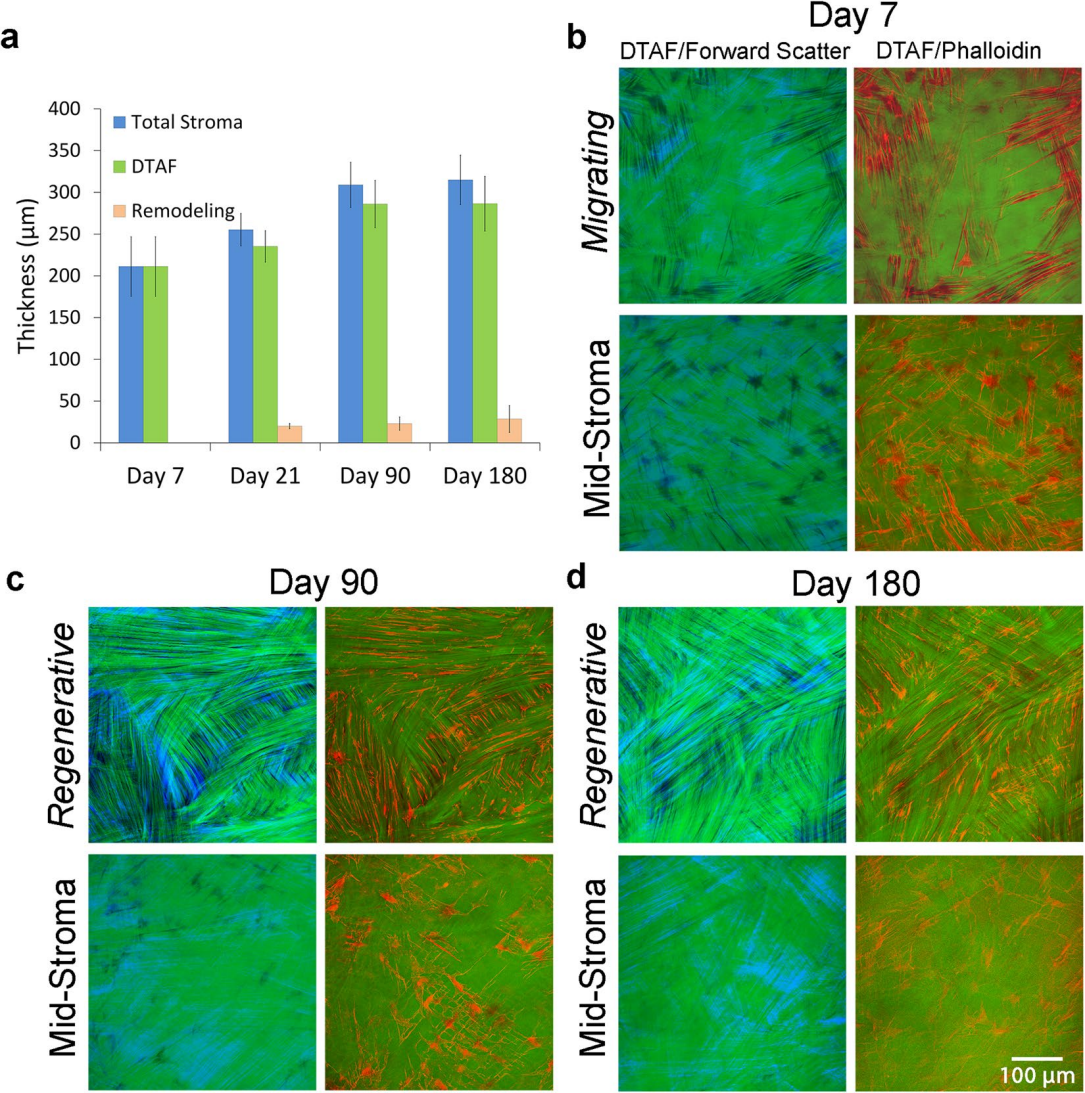
Patterning during regeneration of stromal tissue. (**a**) Measurements for *remodeling* region thickness, thickness of DTAF-labeled stromal tissue, and total stromal thickness over time. Overlays for DTAF (green), forward scatter (blue), and phalloidin (red) en face images at (**b**) 7 days, (**c**) 90 days, and (**d**) 180 days.

To determine the location of stromal re-growth after PRK, the thickness of the *fibrosis*/*remodeling* (non-DTAF) region, DTAF-labeled stroma (which also included the *regenerative* region), and total stroma were compared. As shown in Fig. 7a, growth from the *remodeling* region did not change significantly after forming at day 21; however, total stromal growth progressively increased between day 7 and 180, corresponding to an increase in the thickness of DTAF labeled stroma. *In situ* confocal imaging revealed diffuse DTAF labeling throughout the stroma at Day 7 (shown in Fig. 7b), with the exception of gaps corresponding to stromal cells. In contrast, gaps in the DTAF labeling that did not correspond to stromal cells were consistently observed within the *regenerative* region of the stroma at day 21 (not shown), day 90 (Fig. 7c, top panel), and day 180 (Fig. 7d, top panel). This demonstrated that the gaps contained new collagen secreted post-PRK within the *regenerative* region. In contrast, in the mid-stroma (below the regenerative region), DTAF gaps were filled with cells and not new collagen (Fig. 7b–d, bottom panels) at all time points evaluated.

There was also a transitional area in between the *remodeling* and *regenerative* regions where DTAF labeling was discontinuous; however, forward scatter signaling was ubiquitous throughout the transition region (not shown), indicating that the *remodeling* and *regenerative* regions were interwoven. Within this transitional region, larger gaps in DTAF labeling were filled in with forward scatter signaling that was co-aligned with F-actin labeling (Supplementary Fig. 3). Keratan sulfate labeling was used as a marker for normal ECM protein expression (Supplementary Fig. 4). In the normal unoperated cornea, keratan sulfate (KS) labeling was observed throughout the corneal stroma. However, KS labeling was significantly reduced in the fibrotic region on day 21. At day 90, KS labeling was again observed throughout the stroma (including both the regenerative and remodeling regions).

### Correlation Analysis

**Figure 8:**
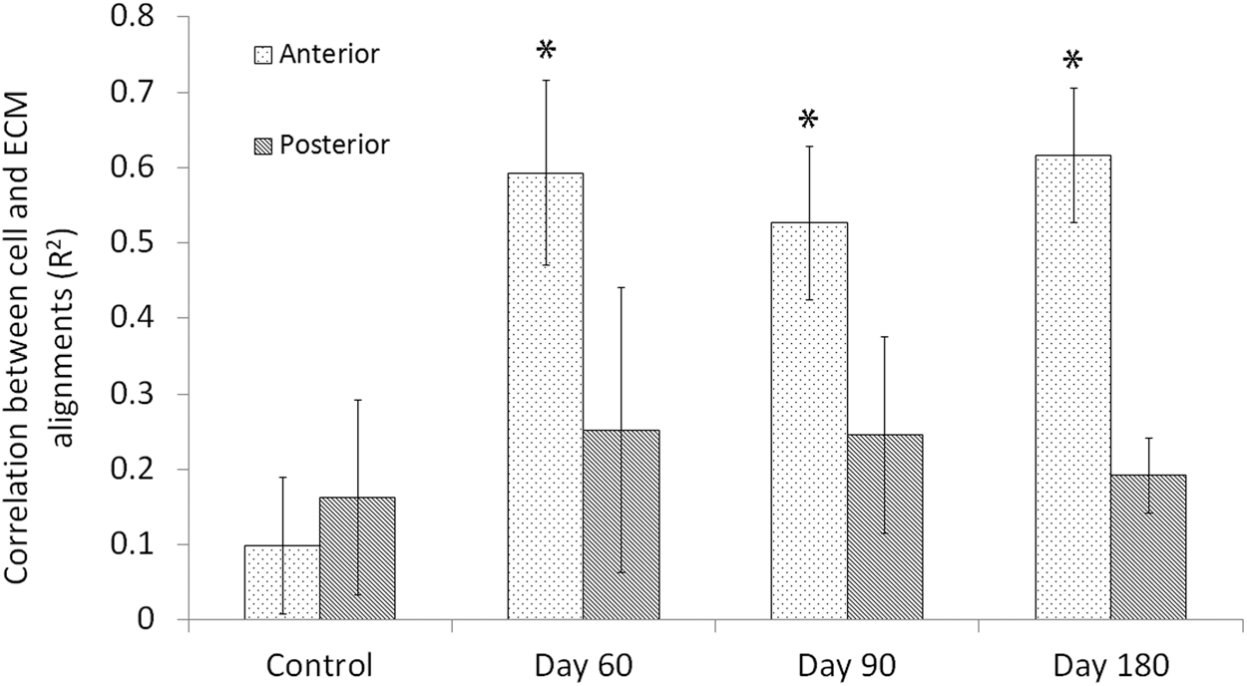
Quantitative analysis of cell/ECM co-alignment in the pre-operative anterior control stroma (control), *regenerative* anterior stroma (day 60, 90, and 180), and posterior stroma. (*P < 0.001, ANOVA, when compared to anterior control stroma).

The co-alignment between cells and collagen was quantified in the regenerative region of the anterior stroma, and in the posterior stroma (Fig. 8). The cells and collagen in the *regenerative* region at each time point had higher correlation (R-squared value) than the posterior stroma and control anterior stroma. These values were consistent with our qualitative assessment of SHG and multiphoton images.

## Discussion

Our lab previously investigated cell/ECM patterning during wound healing using a lamellar keratectomy (LK) model, where an anterior segment of the cornea was mechanically removed^21^. Following LK, keratocytes on top of the wound bed differentiated into myofibroblasts, causing haze and fibrosis. By 60 days, the fibrotic matrix was replaced with a more transparent tissue, and partial regrowth (regeneration) of stromal tissue occurred. Additionally, a thin layer of highly elongated cells replaced myofibroblasts by day 60 at the surface of the ablation in the LK model, similar to the cells near the interface between the *remodeling* and *regenerative* layers observed in the current study post-PRK. Despite evidence of fibrotic reversal in the cornea, mechanistic insights were limited in this initial study. For example, stromal regrowth and haze were not calculated at time points beyond 60 days; thus, long-term remodeling and regeneration of stromal tissue could not be assessed. In addition, there was no way to differentiate between native collagen and new collagen secreted post-PRK; thus, the pattern and location of tissue regeneration within the stroma was not determined. In the current study, we used PRK as a model to provide precise photoablation of the anterior cornea, yielding more consistent, clinically relevant results. In addition, rabbits were studied for up to 6 months after surgery, and DTAF was used to distinguish native collagen from new collagen secreted post-PRK. Thus, the current results provide important new insights into the underlying changes in cell and ECM patterning during the migration, fibrosis, remodeling and regenerative phases of wound healing.

We previously studied cell and matrix patterning following transcorneal freeze injury, which involves intrastromal cell migration to repopulate the injured region^22^. Using SHG imaging, we demonstrated that migrating corneal fibroblasts were aligned parallel to the stromal collagen lamellae. After PRK, there was a region of cell death within the stroma under the laser ablation site. Corneal keratocytes repopulated this injured region via intrastromal migration in parallel with the collagen lamellae, similar to the healing response following freeze injury. *In vitro* studies have shown that cell morphology, migration, and phenotype are directly affected by localized substrate topography^30–32^, and corneal fibroblasts have been previously found to co-align to collagen-coated substrates containing ridges and grooves aligned in parallel^33^. Thus, lamellar patterning likely provides topographical cues that modulate intrastromal corneal fibroblast migration following injury.

In contrast to cells migrating within the corneal stroma, cells that migrated on top of the wound bed were arranged randomly, and transformed into myofibroblasts, consistent with previous studies^6,21,34^. We also observed a sharp transition starting from the fibrotic (*fibrosis/remodeling)* region into the non-fibrotic (*regenerative*) region of the stroma. Cell phenotype drastically changed from myofibroblasts on top of the ablation to elongated, non-fibrotic cells within the native stroma beneath the ablation. DTAF labeling confirmed that there were no myofibroblasts within the native stroma. Several factors may inhibit myofibroblast transformation within the native stroma. One possibility is that the stromal ECM provides topographical cues that prevent cell transformation, since aligned surface grooves have been reported to inhibit the transformation of corneal fibroblasts to myofibroblasts *in vitro*^33^. Additionally, cells residing in between the ECM may be protected against penetration of fibrotic-inducing growth factors, like transforming growth factor-beta (TGF-β) and platelet derived growth factor (PDGF), derived from tears and the epithelium^2,4,35–43^. Finally, biochemical or matrikine signaling from other ECM components could inhibit myofibroblast transformation.

Myofibroblasts were most prevalent at 21 days, which was also when maximum haze was observed qualitatively with slit lamp imaging. Myofibroblasts also have decreased production of crystallin proteins and altered proteoglycan expression, which are both important for maintaining corneal transparency^39,44^. TGF-β induces myofibroblast transformation in the cornea and suppresses interleukin (IL)-1, a cytokine that stimulates myofibroblast apoptosis; therefore, allowing longer time for myofibroblast-derived secretion of fibrotic proteins, like fibronectin and type I collagen, to occur^45–51^. Our experiments revealed maximum secretion of fibronectin at day 21, which was the time point with the highest calculated haze and myofibroblast accumulation (based on α-SMA labeling) in the anterior stroma.

In this study, we showed long-term resolution of fibrosis in the cornea that corresponded with a reduction of corneal haze toward preoperative levels. By day 60, myofibroblasts in the *fibrosis/remodeling* region were being replaced by more quiescent-appearing cells. Previous *in vitro* experiments by Maltseva and associates demonstrated that fibroblast growth factor (FGF)-1 or FGF-2 can trans-differentiate myofibroblasts into fibroblasts^52^. Additionally, it has been shown that as the epithelial basement membrane (EBM) re-forms after injury, there is a drop in TGF-β in the stroma^53^. When TGF-β levels decrease in the wound environment, IL-1 levels increase, which may be signaled in an autocrine or paracrine manner from the myofibroblasts, and induce myofibroblast apoptosis^54,55^. Previous studies have detected myofibroblast apoptosis using TUNEL (terminal deoxynucleotidyl transferase-mediated dUTP nick end labeling) assays starting at 1 month after myopic PRK correction, which falls into the time frame of myofibroblast disappearance presented in this study^56^. It should be noted that the rabbit cornea is thinner than the human cornea and lacks a Bowman’s layer^57,58^.

By day 90, fibrosis and haze were significantly reduced, and interwoven collagen was detected throughout most of the regenerative region by SHG imaging. Although the precise mechanism by which fibrotic ECM is cleared from the anterior stroma has not been described fully, it is likely that a combination of cell-secreted matrix metalloproteinases (MMPs) and disappearance of myofibroblasts (eliminating further deposition of fibrotic ECM) helps remove and eliminate further fibrosis^38,59,60^. The interwoven collagen organization observed from day 90–180 is similar to that observed in the subepithelial region of normal rabbit cornea. Interweaving of collagen lamellae in the anterior stroma has been described as a provider of shear strength against local environmental stresses, preventing sliding and bending of corneal sub-layers and distributing tension between lamellae^10,61–64^. The interwoven pattern of the subepithelial cornea may also be derived from an evolutionary role of maintaining corneal shape for reliable, consistent light refraction in vertebrates^65–67^. We can speculate that a combination of cell contractility, cell/ECM interactions, and environmental tension, forces, and pressure occurring in the eye may play an important role in the development and remodeling of interwoven collagen. It should be noted that the ECM in the remodeled region still produced slightly increased backscatter (haze) as compared to the normal cornea, even at 180 days after surgery.

Keratan sulfate labeling was used as marker for normal ECM expression^68,69^. In the unoperated cornea, keratan sulfate (KS) labeling was observed throughout the corneal stroma. Labeling was significantly reduced in the fibrotic region on day 21, consistent with previous studies showing that myofibroblasts have reduced expression of normal ECM components (including KS)^70,71^. At day 90, KS labeling was again observed throughout the stroma (including both the regenerative and remodeling regions), supporting our conclusion that the cells were producing a more normal ECM as compared 21 days.

Although cells in the *remodeling* region on top of the ablation produced collagen after PRK, the majority of stromal growth occurred in the regenerative region under the wound. Moller-Pedersen *et al*. also reported additional tissue growth after PRK below the ablation region of the stroma, using 3-D reconstructed *in vivo* confocal scans and cross-sectional DTAF images^20,28^. While ECM synthesis during tissue regrowth is regulated by a cascade of cellular biochemical events, the location, organization and patterning of these proteins into the collagen lamellae is equally important in determining the structure, transparency and mechanical properties of the tissue produced. In this study, we provide insights into the mechanisms for tissue re-growth below the ablation surface by using en face SHG/DTAF overlay images. Specifically, during stromal regeneration, we identified gaps in the DTAF labeling that were not co-localized with cells. Interestingly, SHG signaling was observed within these gaps, indicating the presence of newly-secreted collagen. This new collagen was co-aligned both with the native collagen lamellae and elongated corneal keratocytes. Stromal cells have been previously shown to align in the direction of their secreted ECM, and external mechanical tension plays an important role in mediating and stabilizing the orientation of collagen assembly^72,73^.

We also observed punctate F-actin labeling that co-aligned with collagen in both the *remodeling* and *regenerative* regions, which is a hallmark of cell-matrix mechanical interactions^27,74,75^. Cell contractility has been shown to influence non-cell-derived collagen patterning based on forces imposed on the local ECM environment in *in vitro* studies^76–78^. Additionally, the correlation between collagen and fibroblasts in the *regenerative* region may be due to cell elongation in parallel to greatest ECM stiffness within the tissue, as seen with prior studies using corneal fibroblast culture^79^. In the developing chick cornea, keratocytes in the secondary stroma have been noted to have protrusions called “keratopodia” that are co-aligned with collagen, and it has been suggested that these cells use guidance from the collagen lamellae of the primary stroma during the development of the secondary stroma^80–83^. Taken together, the data suggests that cells in the *regenerative* region secrete and organize collagen in a pattern determined by the existing collagen template.

Following freeze injury, stromal tissue is not removed; thus, there is no growth or regeneration of stromal tissue. In this model, aligned fibroblasts in the stroma revert back to a stellate, normal quiescent morphology by day 28^22^. In contrast, following PRK, cells remained elongated and co-aligned with collagen and showed punctate F-actin labeling even at 180 days after injury. After keratectomy, mechanical stresses from intraocular pressure are redistributed throughout the stromal matrix, with a larger load imposed on the stromal ECM due to a decreased thickness^10,11^. It is interesting to speculate that these persistent changes in the mechanical environment of the stroma may provide signals that contribute to long-term cell activation following PRK. In addition, other factors, such as potential persistence of soluble growth factors in the stroma, could also be responsible.

The magnification used for confocal imaging *in vivo* and *in situ* is low enough to allow quantification of sublayer thickness, and cell/ECM backscatter and alignment, but still high enough to appreciate changes in cytoskeletal organization at the subcellular level. Thus this work provides novel insights as compared to previous studies obtained using high magnification techniques (e.g. EM) or cross-sectional histological imaging. Overall, our studies show that an initial fibrotic response occurred in the *fibrosis/remodeling* region of the stroma, which was characterized by significant corneal haze, myofibroblasts, and fibronectin deposition that was organized randomly. Over time, corneal transparency and thickness were restored, and collagen in the *fibrotic* region was remodeled into a more normal, interwoven pattern, possibly due to cell/ECM interactions. Cells and ECM were co-aligned in the *regenerative* region, and stromal growth came primarily from this area. F-actin organization in the *regenerative and remodeling* regions suggests that cell/ECM mechanical interactions likely play a role in aligned collagen deposition and organization. Overall, collagen lamellae appear to provide a template for fibroblast patterning during migration, regeneration and remodeling during corneal wound healing.

## Materials and Methods

### Animals

Experiments in this study were performed using 32 young (3–4 months old) New Zealand White Rabbits (20 females, 12 males; 2.2–3.1 kg; Charles River Laboratories, Wilmington, MA, USA). All animal procedures were in compliance with the ARVO (Association for Research in Vision and Ophthalmology) Statement for the Use of Animals in Ophthalmic and Vision Research and approved by the University of Texas Southwestern Medical Center Institutional Animal Care and Use Committee (IACUC).

### Photorefractive Keratectomy (PRK)

Rabbits were anesthetized using 2% isoflurane gas. Local anesthesia was provided using one drop of preserved 0.5% proparacaine hydrochloride (Alcon Laboratories, Ft. Worth, TX, USA) in the affected eye. The corneal epithelium was removed using laser ablation, and PRK was performed using a VISX Star S4 IR Excimer Laser (Abbott Laboratories, Inc., Abbott Park, IL, USA). Rabbits received a monocular 8-mm diameter, 9.0 diopter PRK myopic correction, creating an approximate stromal ablation depth of ~118 µm. In a subset of rabbits, 5([4,6-dichlorotriazin-2yl]-amino)fluorescein (DTAF; Sigma, St. Louis, MO) was administered directly after PRK to track collagen deposition in the wound bed spatially and temporally as previously described^28,84^. After PRK, rabbits were given 0.3 mg/kg of buprenorphine SR (slow release) subcutaneously, and one drop of preserved 0.3% gentamicin sulfate (Akorn Inc., Lake Foret, IL) was administered for 7 days following surgery in the affected eye.

### *In Vivo* Confocal Microscopy

Rabbits were monitored using a modified Heidelberg Retinal Tomograph with Rostock Corneal Module (HRT-RCM; Heidelberg Engineering, GmBH, Dossenheim, Germany) *in vivo* confocal microscope with Confocal Microscopy Through Focusing (CMTF) software as previously described^22,85^. Rabbits were anesthetized using an intramuscular injection of 50 mg/kg ketamine and 5.0 mg/kg xylazine cocktail, and locally anesthetized with 1 drop of proparacaine. Rabbits were scanned 1 week before PRK (Pre-Op), and at 3, 7, 21, 60, 90, and 180 days after PRK. Since the reflection from the Tomocap can obscure images of the superficial epithelial cells, a thin PMMA (poly(methyl methacrylate)) washer was placed on the Tomocap as previously described^86^. The objective was positioned on the cornea to create a flat field-of-view image in the central cornea. CMTF scans were collected by starting the scan in the anterior chamber and finishing above the epithelium with a constant speed of 60 μm per second. Images were acquired with the rate set to 30 frames per second. To allow quantitative assessment of haze, scans were collected using a constant gain setting, by unchecking the “auto brightness” box in the HRT software interface. Each scan was conducted using a gain of 6^21^. At least 3 scans were collected within the central area of the cornea where PRK was performed. The center of the wound was located by first moving the objective around the cornea to find the borders of the ablated tissue. Each CMTF scan contained a 3-D stack of 384 × 384-pixel images (400 × 400 μm), with a step size of approximately 2 μm between images. Additional scans were collected closer to the wound edge in some animals. In some cases, a portion of the scan was saturated when using manual gain settings due to strong cell/matrix reflectivity. In these cases, additional scans were taken using a gain of 0 so that changes in cell patterning and morphology could be documented. Only the scans taken with a gain of 6 were used for quantitative analysis. In a subset of four rabbits, slit lamp photos were obtained prior to confocal imaging for qualitative analysis of corneal haze (BM 900, Haag-Streit, Bern, Switzerland).

After image acquisition, CMTF scans were saved as “.vol” files, which could be opened into our in-house software to analyze the 3-D changes in cell morphology and cell/ECM reflectivity^85^. The program generates an intensity vs. depth curve, corresponding to the average pixel intensity of each image and the z-depth of that image within the scan, respectively. The relative amount of backscatter, or haze, associated with the stromal keratocytes and ECM was measured by taking the area under the curve between the location of the basal lamina peak (or top of the stroma) and the endothelial peak. A baseline of 13 was chosen for haze calculations, since this value was below the baseline intensity for the normal stroma and above the intensity of the anterior chamber. The thicknesses for the epithelial and stromal layer were also calculated by the CMTF program using the interfaces (peaks on CMTF curve) between each layer in the cornea.

### *In Situ* Multiphoton Fluorescence and Second Harmonic Generation (SHG) Imaging

At 7, 21, 60, 90, and 180 days, a subset of rabbits was sacrificed, and corneas were fixed using anterior chamber perfusion for 5 minutes with a PBS solution containing 1% paraformaldehyde, 5% dextran, 1% dimethyl sulfoxide, and 1% Triton-X-100^74^. Corneas were dissected out of the eye, and fixed for an additional 15 minutes. Next, corneas were washed with 1X PBS twice for 10 minutes each, and labeled *in situ* with either Alexa Fluor 488 or 546 phalloidin (1:20, Molecular Probes, Thermo Fisher Scientific, Waltham, MA, USA) at 37 °C for 3 hours. The corneas were then washed with 1X PBS three times for 30 minutes each. Corneas were then labeled for nuclei with a PBS solution containing Syto (1:1000, ThermoFisher, Waltham, MA, USA) and RNase, DNAase-free (1:100, Roche, Basel, Switzerland) for 1 hour at room temperature.

After labeling, corneas were placed in a glycerol:PBS solution (2:1) overnight to reduce swelling. Next, tissues were imaged using a laser scanning confocal microscope (Leica, SP8, Heidelberg, Germany) with multiphoton and SHG components, and using a 25x water immersion objective (0.95 NA, 2.4 mm free working distance). The central cornea was blocked out using a single edge blade, which allowed for isolation of the injured area, and placed epithelial side down for en face imaging. To capture SHG and multiphoton fluorescence images, a wavelength of 880 nm was used (Coherent Chameleon Vision II, ultrafast Ti: Sapphire laser, Santa Clara, CA). SHG forward scatter, DTAF or phalloidin (F-actin), and SHG backscatter were acquired concurrently, as previously described^21,22^. In DTAF-labeled corneas, phalloidin and Syto were imaged simultaneously in a separate scan.

### Alignment and Correlation Analysis

Cell and/or matrix alignment from *in situ* laser confocal scans were quantified using an in-house MATLAB (MathWorks, Natick, MA, USA) program, which uses a Fourier Transform algorithm to determine the percent of image content aligned at each radial angle within the image^21^. Specifically, original en face images (1024 × 1024 pixels) were divided into 16 sub regions (256 × 256 pixels), as previously described^21,87^. Regions for analysis were selected approximately 30 µm posterior to the basal lamina (anterior stroma) and approximately 65 µm anterior to the endothelium (posterior stroma). A Welch window was then applied to prevent edge effects in the discrete Fourier Transform (FT). The FT power spectrum of each image sub region was calculated, and the average intensity of each angle was calculated from the pixels that lied on the angle and those nearest to the angle in the transformed image. Using polar coordinates, line averages from the center to the periphery of the rotated FT power spectrum were calculated along the radial direction. Points close to the center of the FT power spectrum were excluded from the line averages because they represented low-frequency information (such as shading), which is not of interest. Plots showing both cell and matrix directionality were generated from *in situ* F-actin and SHG images to allow direct comparison of the angle distributions. Regions that lacked cells or SHG collagen signal were excluded from final calculations.

### Immunocytochemistry

After fixing, a subset of corneas were embedded in optimal cutting temperature (OCT) solution, snap-frozen in liquid nitrogen, and stored at −80 °C prior to sectioning. Using a Cryostat (CM3050 S, Buffalo Grove, IL, USA), sections of 7 µm in thickness were cut and mounted onto slides for labeling.

For fibronectin labeling, primary goat anti-human fibronectin antibody (1:200, Santa Cruz, Dallas, TX, USA) was added to the slides and incubated at 37 °C for 2 hours. Sections were then washed three times, 20 minutes per wash, and then incubated with secondary donkey anti-goat FITC- or TRITC-conjugated antibody (1:200 or 1:150) at 37 °C for 1 hour. For α-smooth muscle actin (α-SMA) labeling, primary mouse α-SMA antibody (1:200, Sigma, St. Louis, MO, USA) was added to the slides and incubated for 2 hours at 37 °C. Sections were then washed three times, 20 minutes per wash, and then incubated with secondary goat anti-mouse FITC- or TRITC-conjugated antibody (1:200 or 1:150) at 37 °C for 1 hour. For keratan sulfate labeling, primary mouse anti-keratan sulfate antibody (1:200, Santa Cruz, Dallas, TX) was added to the slides and incubated for 2 hours at 37 °C. Sections were washed with PBS 3 times, 20 minutes per wash. Sections were incubated with FITC-conjugated goat anti-mouse secondary antibody (1:200, Jackson ImmunoResearch Inc., West Grove, PA) for 1 hour at 37 C. For double labeling with F-actin, slides were incubated with Alexa Fluor phalloidin 488 or 546 (1:100) simultaneously with the secondary antibodies. Images were captured using a fluorescent microscope with a 20x objective lens (Leica, DMI3000B, Buffalo Grove, IL, USA).

### Statistics

SigmaPlot (version 12.5; Systat Software, Inc., San Jose, CA, USA) was used for statistical analysis. Linear regression analysis was used to determine the correlation coefficients between the angular distributions of F-actin and the forward scattered SHG signals, as previously described^21^. One way analysis of variance (ANOVA) was used for comparing sub-layer thickness, haze values, and correlation coefficients. Post-hoc analysis using the Holm-Sidak method was used for comparisons between groups.

## Electronic supplementary material

Supplemental Figures
