# Supplementary material for: Assessment of Corneal Stromal Remodeling and Regeneration after Photorefractive Keratectomy
Source: Sci Rep. 2018 Aug 22;8:12580. doi: 10.1038/s41598-018-30372-2 (PMC6105640; doi:10.1038/s41598-018-30372-2)
Supplement: Supplementary file 1 — Supplemental Figures [file 41598_2018_30372_MOESM1_ESM.pdf]

Assessment of Corneal Stromal Remodeling and Regeneration after Photorefractive Keratectomy

Pouriska B. Kivanany[1,2], Kyle C. Grose[1], Madhavi Tippani,[1] Shan Su,[1] and W. Matthew Petroll[1,2]

[1]Department of Ophthalmology, [2]Biomedical Engineering Graduate Program, UT Southwestern Medical Center, Dallas, TX

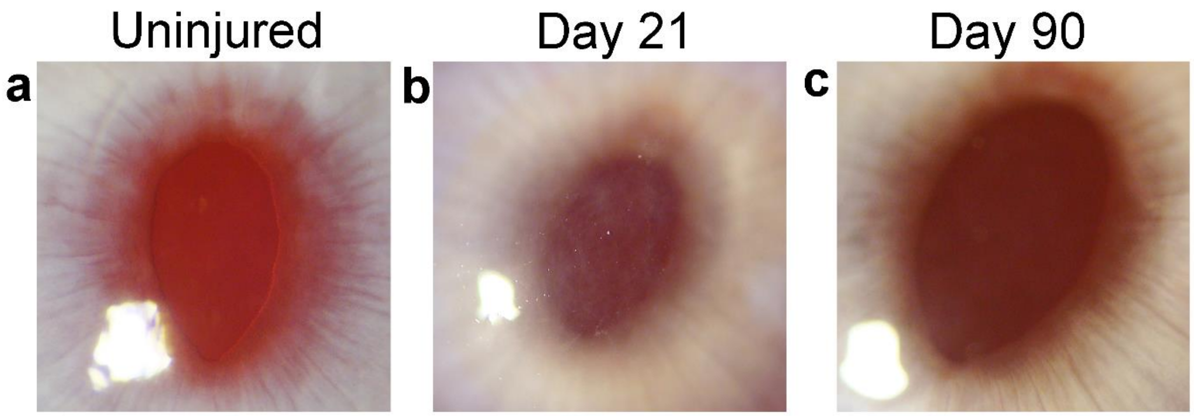

Supplementary Figure 1. Slit lamp images of the central cornea captured pre-operatively (a), and at 21 days (b) and 90 days (c) after PRK.

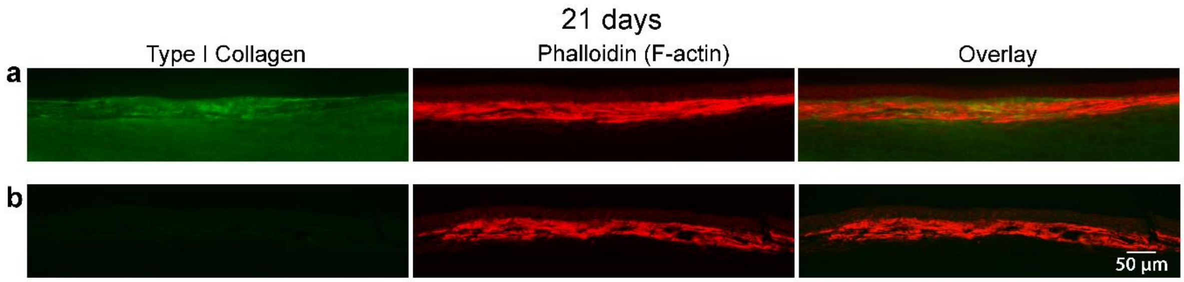

Supplementary Figure 2. Frozen Immunohistochemistry for detection of Type I collagen (1:2000, Gibco (Thermo Fisher), Waltham, MA) and phalloidin (F-actin) in the anterior stroma at 21 days post-PRK. Images are from the anterior segment of the cornea. a) Labeling using both primary and secondary antibodies. b) Negative control using only secondary antibody.

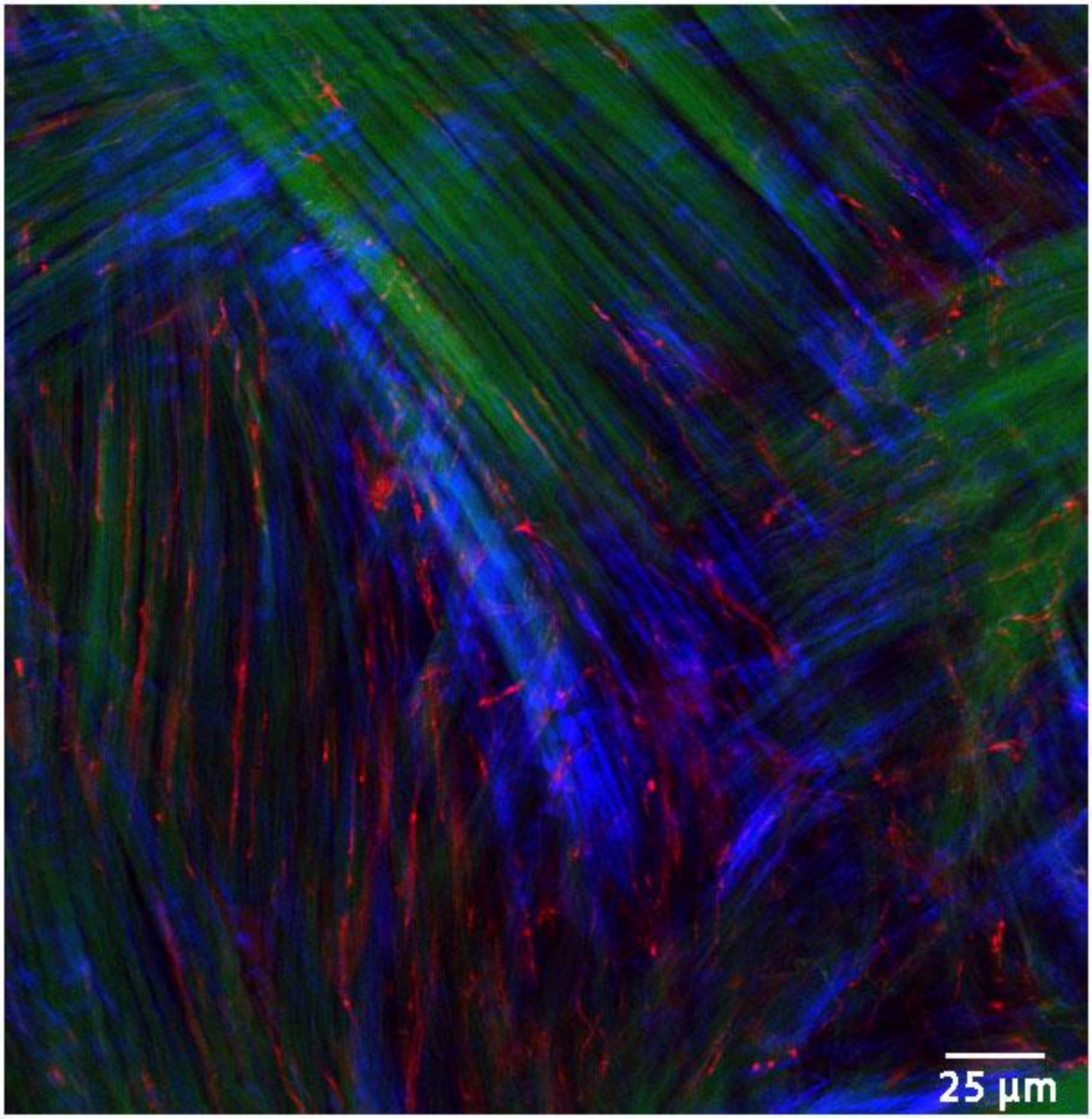

Supplementary Figure 3. Anterior stroma at the interface between the regenerative and remodeling regions at day 90, showing overlay of DTAF (green), forward scatter (blue), and phalloidin (red). Note interactions and coalignment of cells with both native and newly secreted collagen.

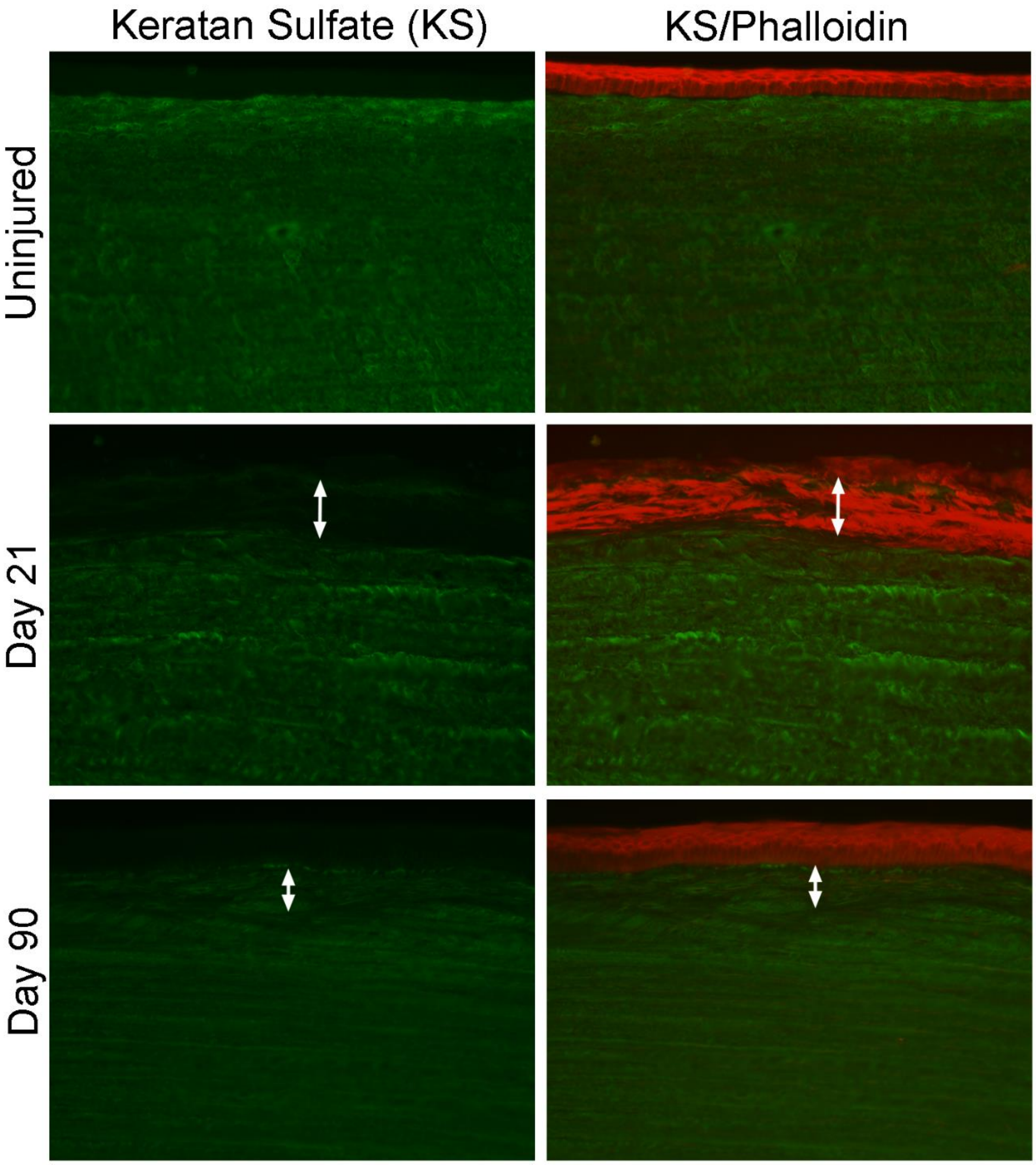

Supplemental Figure 4. Keratan sulfate (green) and F-actin (red) labeling of corneal cross sections. In the normal unoperated cornea, keratin sulfate (KS) labeling was observed throughout the corneal stroma. Labeling was significantly reduced in the fibrotic region on day 21 (arrows). At day 90, KS labeling was again observed throughout the stroma (including the remodeling region, arrows).
